# Death and population dynamics affect mutation rate estimates and evolvability under stress in bacteria

**DOI:** 10.1371/journal.pbio.2005056

**Published:** 2018-05-11

**Authors:** Antoine Frenoy, Sebastian Bonhoeffer

**Affiliations:** Institute for Integrative Biology, ETH Zürich, Zurich, Switzerland; Wageningen Universiteit en Researchcentrum, Netherlands

## Abstract

The stress-induced mutagenesis hypothesis postulates that in response to stress, bacteria increase their genome-wide mutation rate, in turn increasing the chances that a descendant is able to better withstand the stress. This has implications for antibiotic treatment: exposure to subinhibitory doses of antibiotics has been reported to increase bacterial mutation rates and thus probably the rate at which resistance mutations appear and lead to treatment failure. More generally, the hypothesis posits that stress increases evolvability (the ability of a population to generate adaptive genetic diversity) and thus accelerates evolution. Measuring mutation rates under stress, however, is problematic, because existing methods assume there is no death. Yet subinhibitory stress levels may induce a substantial death rate. Death events need to be compensated by extra replication to reach a given population size, thus providing more opportunities to acquire mutations. We show that ignoring death leads to a systematic overestimation of mutation rates under stress. We developed a system based on plasmid segregation that allows us to measure death and division rates simultaneously in bacterial populations. Using this system, we found that a substantial death rate occurs at the tested subinhibitory concentrations previously reported to increase mutation rate. Taking this death rate into account lowers and sometimes removes the signal for stress-induced mutagenesis. Moreover, even when antibiotics increase mutation rate, we show that subinhibitory treatments do not increase genetic diversity and evolvability, again because of effects of the antibiotics on population dynamics. We conclude that antibiotic-induced mutagenesis is overestimated because of death and that understanding evolvability under stress requires accounting for the effects of stress on population dynamics as much as on mutation rate. Our goal here is dual: we show that population dynamics and, in particular, the numbers of cell divisions are crucial but neglected parameters in the evolvability of a population, and we provide experimental and computational tools and methods to study evolvability under stress, leading to a reassessment of the magnitude and significance of the stress-induced mutagenesis paradigm.

## Author summary

The effect of environmental stress on bacterial mutagenesis has been a paradigm-shift discovery. Recent developments include evidence that various antibiotics increase mutation rates in bacteria when used at subinhibitory concentrations. It is therefore suggested that such treatments promote resistance evolution because they increase the generation of genetic variation on which natural selection can act. However, existing methods to compute mutation rate neglect the effect of stress on death and population dynamics. Developing new experimental and computational tools, we find that taking death into account significantly lowers the signal for stress-induced mutagenesis. Moreover, we show that treatments that increase mutation rate do not always lead to increased genetic diversity, which questions the standard paradigm of increased evolvability under stress.

## Introduction

One of the most puzzling and controversial microbial evolution experiments of the 20th century may be the one performed by Cairns and colleagues [1,2] in which *lac*− cells are plated on lactose as the sole carbon source and therefore cannot grow. Revertants toward the *lac*+ genotype continuously appear after plating at a rate and timing seemingly incompatible with the Darwinian hypothesis of selection of preexisting mutants. In the *lac*− construct, the *lacZ* coding sequence is present but nonfunctional, because it is out of frame with the start codon. The *lac*+ revertants are thus frameshift mutants in which this coding sequence is back in frame with the start codon. Most of the controversy initially came from the question of whether these reversion mutations were Lamarckian, in the sense that they would arise at a higher rate when the cells would “sense” that these mutations would be beneficial [3]. However, many additional experiments quickly suggested that this phenomenon can be explained by more standard Darwinian mechanisms, in which genetic changes are not targeted but occur randomly and are then selected or not. While two seemingly conflicting molecular explanations—the stress-induced mutagenesis model and the gene amplification model—emerged, both are conceptually very similar.

In both explanations, mutations occur randomly and independently of their effect on fitness, but the specific conditions of carbon starvation increase the rate at which genetic diversity is generated at the relevant locus (*lacI*-*lacZ* sequence). In the stress-induced mutagenesis model [4], the genome-wide mutation rate is increased as an effect of the stress response triggered by starvation. In the gene amplification model [5,6], random duplications of the *lacI*-*lacZ* system happen before plating on lactose and are then selected in presence of lactose because the frameshift mutation is leaky. A small amount of Beta-galactosidase is still synthesized, permitting cryptic growth due to rare expression errors, which compensate the frameshift. This residual expression becomes higher with more copies of the leaky system. As the copy number of the system increases, a reversion mutation in *lacI*-*lacZ* becomes more likely because of increased target number.

While it is still not clear whether stress-induced mutagenesis is the sole explanation of the phenomenon, the attempts to explain the data presented by Cairns and colleagues have led to a much better understanding of control over mutation rate in response to the environment. An increase in mutation rate under starvation has also been reported in other systems, such as nutrient-limited liquid cultures [7,8] and “aging” colonies on agar plates [9,10]. However, Wrande and colleagues [11] reported that the accumulation of mutants in aging colonies observed by Bjedov and colleagues [10] can be explained by growth under selection without elevated mutagenesis, because the mutation used by the original authors to infer mutation rate is beneficial in these specific environmental conditions.

An emblematic molecular mechanism permitting this regulation of mutation rate is the SOS response (suggested in 1970 [12,13]), in which DNA damage is sensed by bacterial cells and leads to the up-regulation of many genes, permitting mutagenic repair and replication of damaged DNA. While the responsible enzymes were unknown at the time, it has indeed been found subsequently that the SOS response increases the dosage of polIV and polV [14,15]. These error-prone polymerases are able to replicate damaged DNA that the classical DNA polymerase polIII could not replicate, albeit at the price of a higher error rate [16,17]. This strategy, favoring “survival at the price of the mutation,” is only one side of the story. There is a line of evidence suggesting that this higher error rate is not only an unavoidable trade-off with survival. It is also supposed to be a selected property to increase mutation rate under stressful conditions, increasing the chances that one of the descendants obtains a beneficial mutation that makes it able to better withstand the stress [18,19].

The evolution of traits that increase mutation rate under stress needs be considered in the context of second-order selection [20]. Second-order selection relies on the idea that natural selection does not only act on the individual's phenotype and instant fitness but also on its ability to generate fit descendants, leading to selection of properties such as evolvability and mutational robustness [21]. In parallel to the study of environmental control over the mutation rate, genetic determinants of mutation rate have also been studied. It has been shown and is widely accepted that alleles increasing mutation rate (for example, defective mismatch repair or DNA proofreading) can be selected when hitchhiking with the beneficial mutations they permit to generate [22,23]. On the other hand, the possibility of selection of mechanisms increasing mutation rate under stress but not constitutively has been subject to a more philosophical debate [24,25]. While modeling shows such selection is possible [19], it is hard to distinguish whether an observed increase in mutation rate under a specific stress is (i) an evolvability strategy; (ii) an unavoidable trade-off of selection for survival, such as replicating damaged DNA to avoid death at the price of making mutations; or (iii) a direct effect of the stress and not of the stress-response system [26].

This debate is important for a full understanding of the evolutionary relevance of the phenomenon but does not affect the medical implications concerning the risk of de novo evolution of resistance during antimicrobial treatment [27]. Here, we are interested in the general case of mutation rate in growing stressed populations, and we especially focus on antibiotic stress, although our findings may be valid for other biotic and abiotic stresses. It has been suggested that treatment with subinhibitory doses of antibiotics increases bacterial mutation rate, due to induction of various stress-response pathways [28–32]. Many molecular mechanisms underlying this stress response have been elucidated, including the SOS response [29] or the RpoS regulon [30]. Oxidative damage has also been suggested to play a role in antibiotic-induced mutagenesis [28] and death [33–35]. Although still controversial [36], these findings link antibiotic stress to the older question of how bacteria deal with oxidative stress and how oxidative damage impacts mutation rates [37].

However, all the evidence for stress-induced mutagenesis relies on accurately measuring mutation rates of bacteria growing in stressful conditions, and comparing them to those of the same strains growing without stress. Computing such mutation rates under stress is harder than it may seem, because stress may change population dynamics and may thus invalidate the assumptions made by the mathematical models used to compute mutation rate. For example, in the case of subinhibitory concentrations of antibiotics in which net population growth is positive, death may nevertheless happen at a considerable rate. Death events, however, are not detected by standard microbiology methods and are not taken into account by the mathematical tools used to compute mutation rate [38–40].

Indeed, such tools take as inputs only the number of observed mutants at a chosen locus and the final population size, making the underlying assumption that there is no death and that population size is thus sufficient information to summarize growth dynamics. The final population size is used to infer the number of DNA divisions leading to the final observed population from a small initial inoculum. If there is death, more divisions are needed to reach this population size, thus giving more opportunities to acquire mutations. The mutation rate will then be overestimated, because the number of DNA replications will be underestimated.

In this work, we developed an experimental system to compute death rates in populations growing under stress and a computational method to compute mutation rates from fluctuation assays under stress using the computed death rates. We applied this framework to re-estimate mutation rates of *Escherichia coli* MG1655 growing under sub- minimal inhibitory concentration (MIC) doses of kanamycin (an aminoglycoside acting on protein synthesis [41]), norfloxacin (a fluoroquinolone acting on the DNA-gyrase complex and potentially leading to the creation of DNA breaks through the cell machinery [42]), and hydrogen peroxide (an oxidizing agent producing reactive oxygen species that directly affect DNA independently of the cell machinery [43,44]). All these antimicrobials have previously been reported to significantly elevate mutation rate [28,31]. We find the same pattern when computing mutation rate without taking death into account. However, for norfloxacin and kanamycin, the estimated increase of mutation rate due to treatment is strongly reduced after conservatively correcting for death. There remains no signal of stress-induced mutagenesis in the case of kanamycin. These findings confirm our suspicion that neglecting death leads to substantial overestimation of mutation rate under stress.

We also show that mutation rate estimation does not only present experimental and mathematical challenges; it is also not the most relevant measure of evolvability, meaning the capacity of a population to generate adaptive genetic diversity. Indeed, some of the studied subinhibitory treatments cause a significant drop in population size due to both bactericidal and bacteriostatic effects and thus lead to a smaller genetic diversity despite a higher mutation rate. Ironically, evolvability, approximated as the generation of genetic diversity, can be much more easily estimated from experimental data than mutation rate. In our experiments, antibiotics and hydrogen peroxide have very different effects on evolvability: both subinhibitory norfloxacin and kanamycin treatments significantly reduce it, while hydrogen peroxide treatment strongly increases it.

## Results

### Mutation rates are overestimated when neglecting death

**Fig 1:**
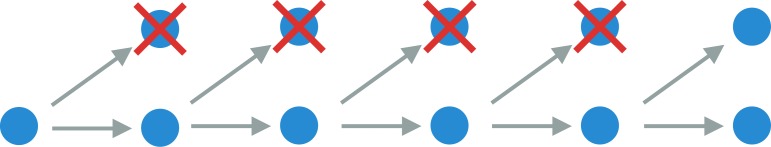
Example with death rate 0.8. One cell division “detected” by change in population size actually requires five “real” cell divisions. Each of these “hidden” DNA replications gives extra chances to acquire a mutation.

Subinhibitory treatments are not necessarily sublethal, because minimal inhibitory concentration is defined at population scale. An antimicrobial treatment is subinhibitory if the population grows (i.e., colony-forming unit [CFU]/mL increases or, more crudely, culture tubes inoculated at low density are turbid after 24 h). However, the death rate can be high, as long as the division rate is higher. Such death events will not be visible to the observer if only population size (CFU/mL) is tracked over time (Fig 1). To reach a given observed final population size, the number of cell divisions has to be higher if there is death. This means that when computing mutation rate using the classical approach (described in the Materials and methods), the number of cell divisions will be underestimated. This is because it is implicitly assumed that there is no death and thus that the final population size is a good approximation for the number of cell divisions. The mutation rate, computed as the number of mutational events divided by the number of cell divisions, will then be systematically overestimated.

**Fig 2:**
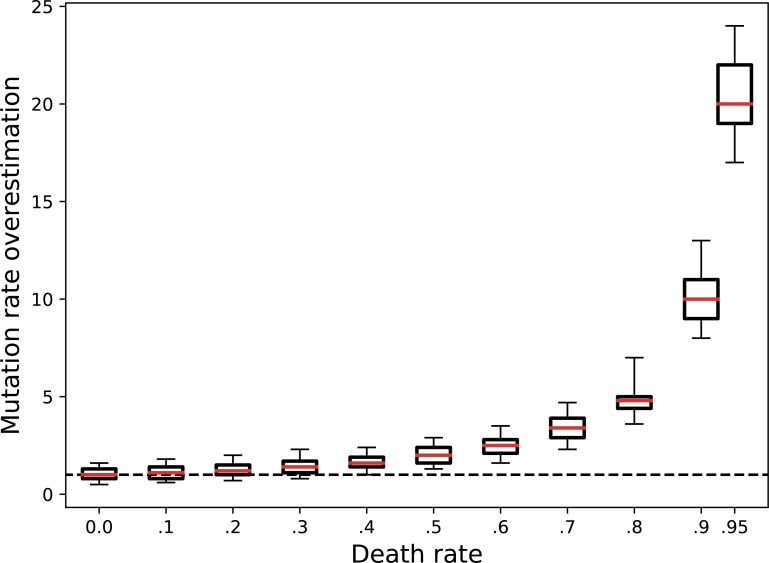
Overestimation of mutation rate when there is death. From simulations of population growth with known death and mutation rate, we estimate the mutation rate using the classical method, which does not take death into account. For each death rate between 0 and 0.95, 1,000 simulations with 24 parallel cultures were performed. For each simulation, we plot the ratio between the computed mutation rate (based on the number of mutants in the final state of the simulations) and the true mutation rate (used as input of the simulations, here 1*10^−9^ per division). The red lines indicate the median values, the boxes indicate the upper and lower quartiles, and the vertical bars indicate the upper and lower 5 percentiles. Underlying data have been uploaded to Zenodo (10.5281/zenodo.1211765).

The above statement—that mutation rates are systematically overestimated when there is death—is the first intuition motivating our work. We explore this intuition more rigorously (Fig 2) using a simulation approach. For an arbitrary chosen value of mutation rate toward a neutral arbitrary phenotype, we simulate the growth of a population of bacteria inoculated from a small number of nonmutant cells with a chosen constant death rate and track the number of mutant and nonmutant cells. We then compute the mutation rate based on the final state of these simulations, using the standard approach (i.e., the fluctuation test as described in the Materials and methods) to test whether we recover the true value of the mutation rate. As shown in Fig 2, the mutation rate is systematically overestimated when there is death, and the higher the death rate, the higher the overestimation. This result is unchanged when varying other population growth parameters, such as the initial and final population sizes, the mutation rate, and the plating fraction (underlying data have been uploaded to Zenodo 10.5281/zenodo.1211765).

### Population dynamics and death in sub-MIC treatments

In the previous section, we show that it is necessary to take death into account when computing mutation rate. For this, tracking population size (and thus net growth rate) during antibiotic treatment, as classically done by plating and counting colony forming units, is not sufficient. It is not possible to know whether a decreased net growth rate in the treatment compared to the untreated control is due to a purely bacteriostatic effect (i.e., the population grows more slowly, but without death) or to a bactericidal effect (i.e., the bacteria keep dividing, potentially at the same rate as without antibiotic, but also die). The first scenario will have no effect on the accumulation of mutants as a function of population size, while in the second scenario, turnover implies a higher number of DNA replications and thus more mutants for a given population size, as explained above.

To disentangle these two effects, we designed a method allowing us to compute growth rate and death rate simultaneously using a segregative plasmid. The segregation dynamic allows us to estimate the number of bacterial cell divisions. Combining this information with the change in population size allows us to estimate growth rate and death rate, as explained in the Materials and methods.

**Fig 3:**
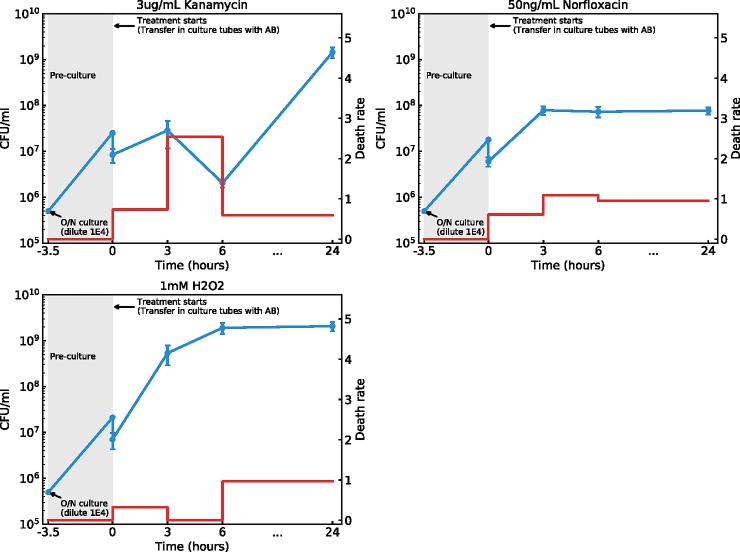
Growth and death dynamics of populations treated with sub-MIC antimicrobials. Each panel shows data for treatment with a different antimicrobial. Blue dots (left axis) joined by straight blue line represent population sizes measured by plating, expressed as CFUs per mL of culture. Red line (right axis) represents relative death rates, computed from plasmid segregation data, corresponding to the average number of death event per division event between two successive time points. For each treatment, the behaviors of at least 4 fully independent biological replicates, performed on different days with different batches of medium and comprising at least 4 replicate populations, are averaged. Error bars indicate standard error of the mean between biological replicates. Death rates higher than 5 were set to 5. See also S1 Supporting Information for a representation of all biological replicates. Underlying data have been uploaded to Zenodo (10.5281/zenodo.1211765). CFU, colony-forming unit; MIC, minimal inhibitory concentration.

Our ultimate goal is to reliably estimate mutation rates of bacteria treated with subinhibitory doses of antimicrobials. To this end, we quantify population dynamics and compute mutation rates toward a chosen neutral phenotype (resistance to rifampicin, conferred by substitutions in the gene rpoB) in populations exposed to subinhibitory doses of other antimicrobials. Our mutagenesis protocol is inspired by the standard fluctuation test with additional measurements of plasmid segregation to compute death rate, as detailed in the Materials and methods. The population dynamics are quantified as a combination of two variables: CFU at various time points (e.g., 0 h, 3 h, 6 h, and 24 h after treatment starts) and relative death rate (compared to birth rate) between pairs of two successive time points. We represent these population dynamics in Fig 3 for the chosen subinhibitory antimicrobial treatments. We use kanamycin at 3 ug/mL, norfloxacin at 50 ng/mL, and hydrogen peroxide (H_2_O_2_) at 1 mM, allowing direct quantitative comparisons with the data from Kohanski and colleagues [28]. We also include untreated populations, in which we assume there is no death, to fit the plasmid segregation parameters (see Materials and methods).

We find that for norfloxacin, there is a strong death rate in all phases of growth and a strong impact of the treatment on final population size. For H_2_O_2_, death is only detectable in stationary phase and the treatment is mostly bacteriostatic during growth. For kanamycin, the dynamics are more complex, because an initially high death rate leads to a strong decline of population size during the first 6 h of growth, followed by a recovery leading to a final population size close to the one reached in untreated controls. During this second phase of growth following the bottleneck at 6 h, death rate is still substantial. This clearly shows that none of the three studied treatments are fully sublethal and thus that the implicit assumption of no death made when using the standard methods of computation of mutation rate (as done by Kohanski and colleagues [28]) does not apply.

### Mutation rate in sub-MIC treatments

**Fig 4:**
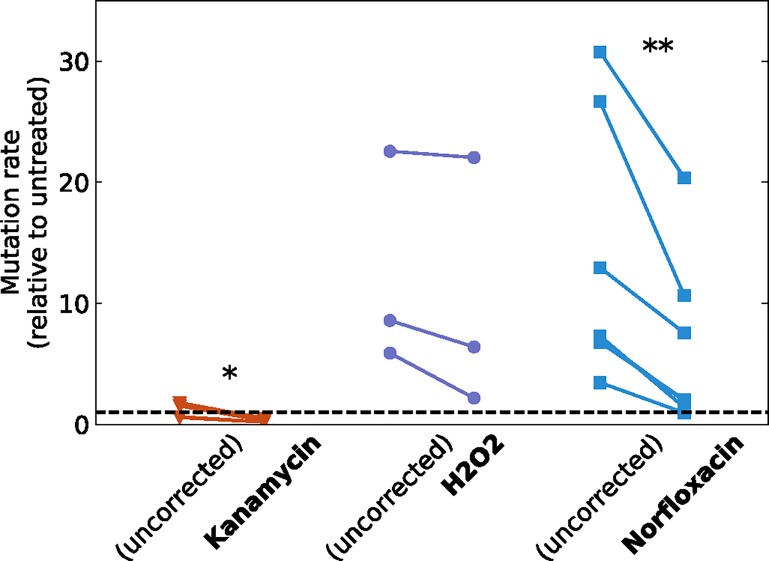
Mutation rate when treating with a sub-MIC dose of kanamycin, H_2_O_2_, or norfloxacin compared to untreated. The uncorrected mutation rate is the one that would be computed based on our data when death is not taken into account. Each point corresponds to a fully independent biological replicate comprising 24 parallel populations. All the computed mutation rates are normalized by the average mutation rate computed in absence of treatment. The horizontal dashed line (*y* = 1) thus indicates the mutation rate of untreated populations. For each treatment, we performed a paired *t* test to estimate whether the corrected mutation rate was significantly lower than the uncorrected one (* *p* < 0.05, ** *p* < 0.01). Underlying data have been uploaded to Zenodo (10.5281/zenodo.1211765).

We developed computational tools to quantify mutation rate, taking into account the measured population dynamics and accounting for death. Our software, ATREYU (Approximate bayesian computing Tentative mutation Rate Estimator that You could Use), is described in the Materials and methods. It takes as input any arbitrary population dynamics, described as a list of population sizes (i.e., CFU/mL for several time points) and an associated list of death rates between pairs of consecutive time points. This input is thus exactly what is shown in Fig 3. We apply this method to analyze the results of our mutagenesis protocol, to quantify whether and by how much subinhibitory treatments with kanamycin, norfloxacin, or H_2_O_2_ increase mutation rate. We show the effect of treatment on mutation rate in Fig 4. We also plot the uncorrected mutation rate estimate, assuming no death as would be obtained by methods such as FALCOR (Fluctuation AnaLysis CalculatOR) [39], bzRates [40], or rSalvador [38]. Clearly, not taking death into account leads to a strong overestimation of the mutation rate for both kanamycin and norfloxacin. In the case of kanamycin, correctly computing the mutation rate removes all signal for stress-induced mutagenesis. In the case of norfloxacin, this signal is strongly lowered, from a 14-fold to a 6-fold increase. For H_2_O_2_, the signal is less affected, which can be attributed to death rate only being significant in stationary phase. This confirms that neglecting death leads to a systematic overestimation of mutation rates and that taking into account the full population dynamics is necessary and leads to significantly different patterns depending on the antimicrobial and its effect on growth and death.

### The link between evolvability and mutation rate depends on population dynamics

The quantification of mutation rate in different conditions is not sufficient to answer the question of whether subinhibitory antibiotic treatments increase evolvability in general and, in particular, increase the likelihood of emergence of a resistant mutant and thus the probability of treatment failure. Indeed, mutation rate is expressed per DNA division, but, as we have shown in the previous section, antibiotic treatment may significantly change the number of susceptible cells and the number of replications that these cells have undergone. Intuitively, if a treatment multiplies mutation rate by 10 but divides population size by 100, it is not likely to lead to an increased genetic diversity. This intuition has also been given by Couce and Blázquez (Fig 2 of [45]) but has been largely ignored in the literature as it was not the main message of this review. Conversely, a treatment that does not affect mutation rate and only slightly affects carrying capacity but causes death and turnover may result in a significantly increased genetic diversity.

**Fig 5:**
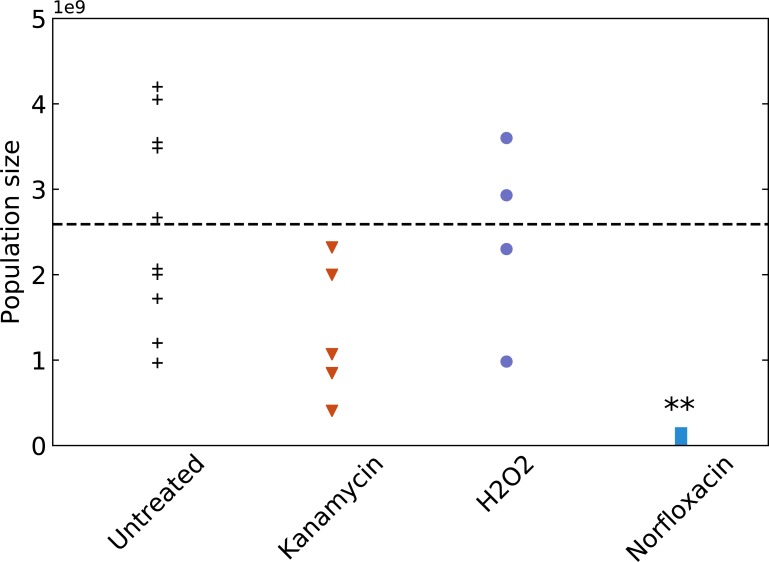
Population size reached after 24 h in treated and untreated conditions. Each point corresponds to a fully independent biological replicate. For each of these biological replicates, average population size is estimated by plating appropriate dilutions of at least 6 replicate populations on nonselective medium. The dashed black line indicates the average size of the untreated populations. We performed an unpaired *t* test to estimate whether treated population sizes are significantly different than untreated ones (** *p* < 0.01). Underlying data have been uploaded to Zenodo (10.5281/zenodo.1211765).

We first show the effect of subinhibitory treatment on final population size in Fig 5. While H_2_O_2_ does not affect final population size, there is a strong effect of 1–2 orders of magnitude for norfloxacin and a significant but smaller effect of around 50% reduction for kanamycin. This supports our intuition that at least for norfloxacin, the few-fold increase in mutation rate we report (Fig 4) is probably uncorrelated to any increase in genetic diversity.

We expect the generation of genetic diversity to depend on (i) the number of cells alive, (ii) the population dynamics of these cells, and (iii) their mutation rate. Addressing the effect of stress on mutation rate as done in the previous section is necessary for a proper understanding of the bacterial stress response and of DNA repair mechanisms. Nevertheless, mutation rate is not the relevant measure to understand the effect of stress on the generation of genetic diversity and thus on evolvability.

As a simple quantification of the generation of genetic diversity and thus an approximation of evolvability, we measure the number of mutants at a neutral locus, here the base-pair substitutions conferring resistance to rifampicin in the gene rpoB.

**Fig 6:**
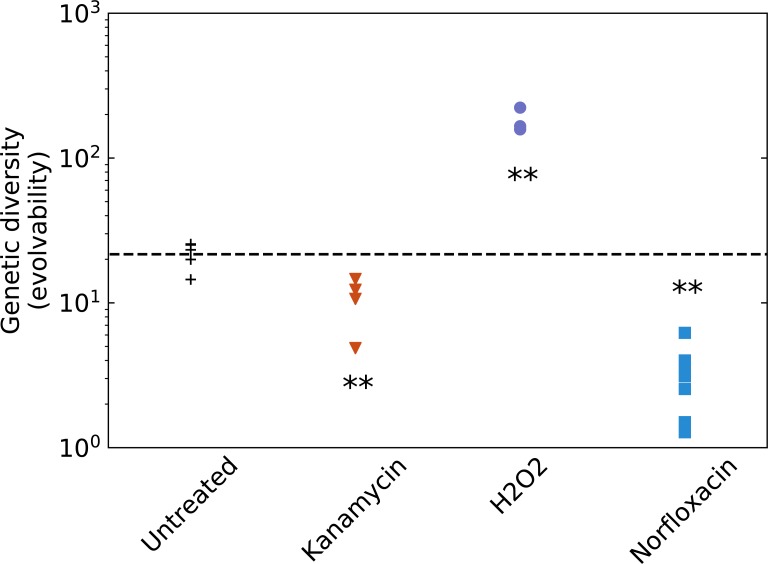
Evolvability of untreated and treated populations. For each treatment, we estimate evolvability as the number of rifampicin-resistant mutants in the population after 24 h of growth. The dashed black line represents the average evolvability of the untreated populations. Each point is a fully independent biological replicate comprising 24 replicate populations in which the number of rifampicin-resistant mutants is averaged. We performed an unpaired *t* test to estimate whether evolvability of treated populations is significantly different than this of untreated ones (** *p* < 0.01). Underlying data have been uploaded to Zenodo (10.5281/zenodo.1211765).

We plot in Fig 6 the absolute number of rifampicin-resistant mutants in the final population for all treatments and for untreated control. Evolvability is reduced by a few-fold by kanamycin treatment (as expected, since this treatment decreases population size without increasing mutation rate). While norfloxacin and H_2_O_2_ both induce a small increase in mutation rate, they interestingly have strongly opposite effects on evolvability. Treatment with H_2_O_2_ increases evolvability by more than one order of magnitude, while treatment with norfloxacin reduces it by a similar amount. This is due to the very different effects these antimicrobials have on population dynamics: while H_2_O_2_ does not affect final population size, norfloxacin causes a strong decrease in population size due to both bactericidal and bacteriostatic effects.

So independently of the question whether antibiotics increase mutation rate, we show that the sub-MIC treatments we studied do not in any way increase evolvability. Thus, the standard rationale, that these subinhibitory treatments would increase the risk of emergence of resistance and treatment failure because of a higher generation of genetic diversity [46], does not hold.

**Fig 7:**
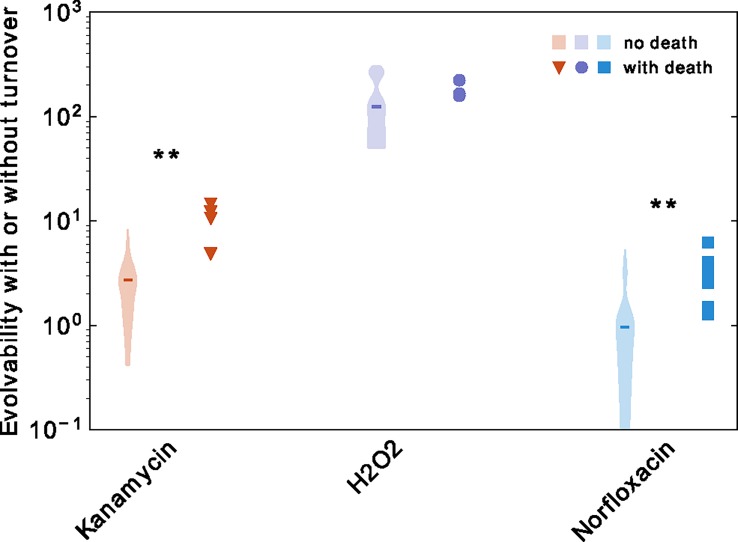
Contribution of turnover to evolvability. For each treatment, we plot the measured evolvability of the populations (red triangles, purple dots, and blue squares) and the estimate that was obtained if the same population size was attained without cell death and with the same mutation rate (violin plots, 100 replicate simulations for each biological replicate). The median of all simulations for a given treatment is represented as a horizontal bar. For each treatment, we performed an unpaired *t* test to test whether the evolvability with death is different than the evolvability without death (** *p* < 0.01). Underlying data have been uploaded to Zenodo (10.5281/zenodo.1211765).

This effect is largely due to a strong reduction in population size, which implies a loss in genetic diversity. Population size and mutation rate are not, however, the only factors affecting evolvability. We may also ask how much the measured turnover in our experiments contributes to evolvability. To answer this question, we simulate the same population dynamics as observed for each treatment but without death: Each population reaches the same final population size as measured in our experiments, with the same mutation rate as computed, but with no death. This is similar to what would happen if the antibiotics only had a bacteriostatic effect. For each simulation, we quantify evolvability using the same measure as previously, i.e., the absolute number of mutants for our phenotype of interest in the final population. We compare this simulated evolvability without turnover with the actual measured evolvability in Fig 7. For kanamycin and norfloxacin, turnover significantly increases evolvability by a few-fold.

### Conclusions

In summary, our results show that (1) mutation rate is systematically overestimated in subinhibitory treatments because of death, (2) mutation rate is not the only parameter that controls the generation of genetic diversity or evolvability, (3) population size and turnover play a key role in evolvability, and (4) treatment with subinhibitory doses of norfloxacin or kanamycin significantly decreases evolvability, measured as the generation of genetic diversity at population scale. These results are in apparent disagreement with the conclusions of previous studies on antibiotic-induced mutagenesis. This discrepancy is due to both miscalculation of mutation rates (which occurs when one neglects the effect of population dynamics) and misconceptions about the link between mutation rate and evolvability in these classical papers.

## Discussion

Understanding genetic and environmental control of evolvability is central for the understanding of microbial adaptation to constantly changing environments. Evolvability is defined as the capacity of a population to generate adaptive genetic diversity. This can be decomposed in two variables: the amount of genetic diversity generated by a population (often inaccurately attributed to the mutation or recombination rate only) and the fraction of this diversity that is adaptive. We are here interested in the former. Genetic control over the amount of generated genetic diversity has been studied for a long time in the field of mutation rate evolution [20,22]. The existence of constitutive mutator alleles in bacteria has been discovered before the mechanisms of DNA replication [47], and the selection pressures leading to their transient increase in frequency have been elucidated through both theoretical and experimental studies [23,48]. Observing the evolution and fixation of such mutator alleles from nonmutator lineages in a long-term evolution experiment [49] plausibly facilitated the acceptance of these theories. On the other hand, plastic, environment-dependent control over the generation of genetic diversity has been a controversial paradigm shift in bacterial evolution [18].

It has been proposed for a long time that various stresses can increase mutation rates in bacteria [9,10], including those triggered by antimicrobial treatments [28–32]. Several molecular pathways have been shown to be implicated in this phenomenon, the emblematic one being the SOS response [12]. In this work, we have shown that the effect of stress on mutation rate can not be computed properly with the existing tools, because the underlying mathematical models make the assumption that there is no stress or, more precisely, that the stress does not affect population dynamics. We develop experimental and computational tools to measure population dynamics and compute mutation rates under stress and apply them to the question of mutagenesis due to antibiotic treatment. We have shown that the intuition that low doses of antibiotics are dangerous because they lead to a higher generation of diversity is based on a misinterpretation of valid experimental data for two reasons: (1) the increase in mutation rate is overestimated due to overly simplistic assumptions, and (2) a higher mutation rate does not lead to a higher genetic diversity if population dynamics are affected (e.g., if population size is reduced).

The question of emergence of resistance alleles due to low doses of antibiotics (reviewed by Andersson and Hughes [50]) cannot, however, be entirely addressed by measuring the generation of genetic diversity. The study of adaptive evolution can be decomposed in two parts: generation of diversity and natural selection acting on this diversity. While we have shown that treatment with a subinhibitory dose of norfloxacin does not increase but rather strongly decreases the amount of generated genetic diversity, it has also been reported that resistance alleles can be maintained and enriched by selection, even at very low antibiotic concentration [51]. Such selection of preexistent alleles may be a much more valid reason for concern about subinhibitory treatments. However, the literature is not as unanimous regarding bacteria residing within a patient with an immune system, rather than in a test tube [52]. It has, for example, been suggested that treating with a lower dose of antibiotics could slow down the selection of existing resistance alleles by decreasing their fitness advantage compared to the sensitive, wild-type strain, without compromising the success of the treatment [53, 54]. Combining our results with these papers calls for a reevaluation of the evolution of antibiotic resistance at low doses of antibiotics.

The question of the potentially adverse effects of low doses of antibiotics has been of longstanding interest in the medical community, as is evidenced by the famous quote from Alexander Fleming's Nobel lecture [55], “If you use penicillin, use enough.” However, given the time of this research (penicillin was discovered in 1928 and thus 15 years before Luria and Delbrück), one should not be surprised that this often cited out-of-context advice relies on a rather Lamarckian reasoning in terms of educating rather than selecting for resistance:

Then there is the danger that the ignorant man may easily underdose himself and by exposing his microbes to non-lethal quantities of the drug make them resistant. Here is a hypothetical illustration. Mr. X. has a sore throat. He buys some penicillin and gives himself, not enough to kill the streptococci but enough to educate them to resist penicillin…

Our findings are also relevant outside of the context of evolution during antibiotic treatment. As we mentioned, mutagenesis in bacteria under nutritional stress was a key development in the understanding of the bacterial stress response and DNA repair, with a recent regain of interest [7,8]. Our experimental system can a priori not be applied to study starving bacteria, for two reasons: (1) our plasmid segregation method only gives sufficient signal in nonstationary populations, and (2) many of the observations on starvation-induced mutagenesis are dependent on the presence of some spatial structure (for example, bacterial colonies on agar plates [2,9,10]). In this second case, the population dynamics become much more complex and are unlikely to be realistically approximated by a single relative death rate parameter. But the exact same questions remain to be elucidated in this field: How many cell divisions happen in these starving colonies? In batch cultures, is stationary phase really stationary, or is there some turnover and recycling as recently suggested [56]? And more importantly, where does death come from: is it an unavoidable, externally caused phenomenon; or is there an internal component, such as an altruistic programmed cell death [57], or just traits selected in other environments that give a maladaptation to certain stresses [58]?

Stress-induced mutagenesis is of interest for several research fields, with different questions. We showed that the relevant question in a clinical setting is not directly about mutation rate but about evolvability and that the link between both is confunded by the effect of treatment on population dynamics.

One the evolutionary side, the central question about stress-induced mutagenesis is “Is it adaptive?” Studying the molecular mechanisms of stress response will shed light on one part of the answer: is the increase in mutation rate controlled by the cell, or is it an unavoidable consequence of the stress? In this regard, the three antimicrobials we study seemingly have very different properties. H_2_O_2_ is creating reactive oxygen species that directly damage DNA independently of the cell machinery, iron being the only necessary catalyst [43,44]. The way DNA damage leads to mutations is controlled by the cell but is more likely to be a consequence of selection for survival (“survival at the price of the mutation”), rather than selection for evolvability. On the other hand, kanamycin acts on protein synthesis [41], and any hypothetical mutagenic effect would thus go through the cell machinery. Norfloxacin is in between, because it acts on the DNA-gyrase complex, leading to an arrest of DNA synthesis and, in some conditions, to double strand breaks [42].

Recent findings from J. Collins and colleagues, however, suggest that these different scenarios are not as distant as they may seem, because they suggest that the production of reactive oxygen species is a feature of all bactericidal antibiotics [33–35]. While supporting the idea that antibiotic treatment increases mutation rate and does so in correlation with bactericidal activity, these debated findings would also suggest that such increase in mutation rate does not stem from selection for evolvability.

In a nutritional stress scenario, Maharjan and colleagues [8] show that at equal effect on growth rate, limitation of different nutrients has very different effects not only on mutation rate but also on mutational spectrum, again showing the need for a mechanistic understanding of the molecular details and suggesting that the evolutionary outcome is much more complex than a linear increase in mutation rate in response to starvation.

We provide tools that may help further developments of these questions. Our software, ATREYU, can be used to compute mutation rates from mutant counts in populations with arbitrary but known birth and death dynamics. The mutant counts are obtained by a protocol similar to the classical fluctuation test. The birth and death dynamics can be obtained by several methods. We used plasmid segregation, but other methods may be possible, such as segregation of engineered self-assembling fluorescent particles [59], isogenic strain tagging [60], Carboxyfluorescein succinimidyl ester (CFSE) membrane staining [61], or direct microscopic observations at single-cell resolution. Microscopic observations with cell tracking may give much more precise and less noisy information than other methods but are only suitable when the death rate is sufficiently low, because only a limited number of cells can be tracked. We believe that death and cell turnover are crucial factors in evolutionary microbiology but are often neglected, in part due to the lack of standard methods to measure them. In immunology, in which the population dynamic of lymphocytes has been recognized as a central question [62], many methods have been developed [63], including the aforementioned CFSE membrane staining.

While our method could be adapted for many nonstandard assumptions other than death (e.g., fitness cost of the mutation or partial plating), it shares some of the limitations of the more classical systems. Firstly, we suppose that each cell is fully monoploid and has only one chromosome and thus that the number of DNA replications is the number of cell divisions. However, some quinolone antibiotics are known to cause filamentation [64], increasing the number of chromosomes per cell [42] and potentially changing the evolutionary dynamics [65]. Further complicating the picture, recent work [66] shows that even within a single chromosome, multifork replication may cause different ploidy levels on different loci, affecting mutation rate estimates and evolutionary dynamics. Secondly, we also consider that time does not matter, in the sense that the probability of mutation per division is independent of the growth rate, and that nondividing bacteria do not accumulate mutations, justifying the expression of mutation rate as a quantity of mutations per division event and not per unit of time. Since Luria’s and Delbrück’s experiment, this has been the standard assumption both on the microbiological and mathematical side [67]. However, recent data on fission yeasts suggest that nonreplicating cells may accumulate mutations at a different rate and spectrum than diving cells [68]. Finally, we make the assumption of homogenous behavior in the population, excluding the possibility that different subpopulations have different death and mutation rates. The question of whether a small subpopulation in a different physiological state may contribute most of the mutational supply is still unresolved. Theoretical work [69] shows that such situation could have a large impact on the evolutionary dynamics.

Zooming out from evolutionary microbiology, mutagenesis research in bacteria shows an interesting parallel with recent advances in cancer research. For a given cell growth dynamic (organogenesis, from stem cells to an organized population of differentiated cells), a higher mutation rate (expressed per cell division) will boost the accumulation of mutations and thus the risks of cancer. This increase in mutation rate can be genetic, such as in the case of hereditary nonpolyposis colon cancer caused by a deficiency of mismatch repair [70], or environmental, such as exposure to carcinogenic compounds [71–73]. All of this is now part of textbook science on cancer and is similar to an increase in mutation rate in a bacterial population due to genetic (mutator alleles [47]) or environmental (stress-induced [18] or stress-associated [26] mutagenesis) factors.

Tomasetti and Vogelstein [74] recently reported that the number of stem cell divisions is a strong predictor of cancer risk per organ. This is in parallel with our findings, which show that the number of cell divisions is central to predict the generated genetic diversity in a population of cells. Tomasetti and Vogelstein caused a major controversy by concluding that cancers would thus mostly be due to “bad luck” (i.e., unavoidable consequences of the large number of cell divisions) rather than to environmental factors (e.g., exposure to mutagenic chemicals). We show here that the generation of genetic diversity depends on both mutation rate and cell population dynamic, which is in line with many studies that have criticized the interpretation of the data made by Tomasetti and Vogelstein.

The challenge of understanding evolvability in bacterial population is thus strikingly similar to the one of understanding cancer, in the sense that the outcome depends on a complex interplay of extrinsic and intrinsic factors acting at different scales. In the case of bacteria, additional complexity stems from the fact that the same treatments may both impact the number of cell divisions (death and turnover) and the mutagenicity of each division. The picture is further complicated by the difficulty of disentangling the direct effects of the drug from the effects of the stress response triggered by the drug. But fortunately, while separating and measuring each factor requires complex experimental methods and mathematical tools, measuring evolvability on neutral loci is simpler, at least in bacteria. We hope that our study will encourage researchers in the field to question more not only the appropriateness of the tools they use for mutation rate estimation and the assumptions implicitely made by using these tools but also the pertinence of the variable they choose to report.

## Materials and methods

### Experimental setup

**Fig 8:**
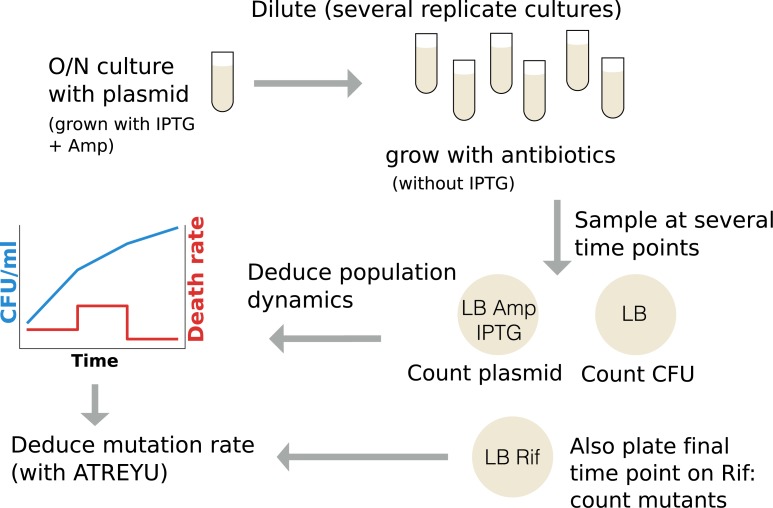
Experimental protocol and data flow. ATREYU, Approximate bayesian computing Tentative mutation Rate Estimator that You could Use; CFU, colony-forming unit.

Our mutagenesis protocol is directly inspired by the one used by Kohanski and colleagues [28] (which is in turn similar to that of Luria and Delbrück [75]) with the inclusion of a segregative plasmid to compute death rate, as explained further below and graphically represented on Fig 8.

A culture of *E*. *coli MG1655* (with plasmid pAM34) is inoculated from a freezer stock and grown overnight in LB supplemented with 0.1 mM IPTG and 100 ug/mL of ampicillin (to ensure maintenance of pAM34). After the culture reaches stationary phase (at least 15 h of growth), it is washed 3 times in normal saline (9 g/L NaCl) to remove traces of IPTG and then diluted 10,000 times in a 500 mL baffled flask containing 50 mL of LB (to maximize oxygenation). After 3.5 h of growth, the culture is inoculated at a ratio of 1:3 in 24 culture tubes containing a total volume of 1 mL of LB supplemented with one of the studied antimicrobials at subinhibitory concentration (3 ug/mL kanamycin, 50 ng/mL norfloxacin, 1 mM hydrogen peroxide, or untreated control). After 24 h of growth at 37°C, the cultures are plated at appropriate dilutions on 3 different LB agar medium: LB only to count the total number of bacteria (CFU), LB supplemented with 100 ug/mL ampicillin + 0.1 mM IPTG to count the number of bacteria bearing a copy of the segregative plasmid, and LB supplemented with 100 ug/mL rifampicin (plated volume: 200 uL) to count the number of mutants toward the phenotype of interest. Additionally to this 24 h time point, cultures are also plated on LB and LB + ampicillin + IPTG at intermediate time points (3 h and 6 h) to have a more accurate quantification of plasmid segregation dynamics and thus a better time resolution for the estimation of death rate. The plates are incubated between 15 h and 24 h for LB and LB ampicillin IPTG and exactly 48 h for LB rifampicin before counting colonies. Further experimental details are given in S5 Supporting information.

### Measuring death using plasmid segregation

**Fig 9:**
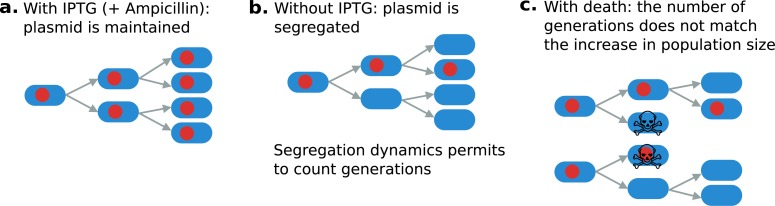
Segregation of a plasmid with inducible replication allows us to estimate death rate. (a) Maintenance of the plasmid in presence of IPTG, (b) segregation of the plasmid in absence of IPTG, (c) computing population dynamics (death rate) from plasmid segregation in absence of IPTG. IPTG, Isopropyl β-D-1-thiogalactopyranoside.

pAM34 is a colE1 derivative whose replication depends on a primer RNA put under the control of the inducible promoter pLac [76]. Under the presence of 0.1−1*mM* IPTG (nonmetabolizable inducer of the lactose operon), the plasmid is stably maintained in every cell. When IPTG is removed from the growth medium, the plasmid is not replicating anymore, or not as fast as the cells divide, and thus is stochastically segregated at cell division. The decrease in plasmid frequency between two time points then allows us to compute the number of bacterial cell divisions that occurred between these two time points. Combined with the change in population size, this allows us to compute average death rate and growth rate between these two time points (see Fig 9 and mathematical explanations below). Such segregation measures have been used in a less quantitative way by other researchers [77,78] to crudely infer overall population turnover in vivo.

pAM34 also carries a betalactamase. The number of plasmid-bearing bacteria can thus be counted by plating an appropriate dilution of the culture on LB supplemented with 0.1 mM IPTG (to ensure maintenance of the plasmid within colonies founded by a plasmid-bearing cell) and 100 ug/mL ampicillin (to only permit growth of colonies founded by a plasmid-bearing cell). The total number of bacteria is determined by plating an appropriate dilution of the culture on LB.

Because mutational dynamic does not depend on time, we chose to compute relative death rate (ratio of death rate and growth rate as functions of time), which is the average number of death events per division event.

The link between plasmid segregation, death, and number of divisions between two time points can be expressed mathematically as follows.

If we have the following:

- *F*: the frequency of cells bearing at least a copy of the plasmid, measured by plating on LB + ampicillin + IPTG;
- *N*: the total number of cells, measured by plating on LB;
- *res*: the rate of residual replication of the plasmid relative to the division rate in absence of IPTG;
- *g*: the number of generations, i.e., the average number of duplications each genome present at final time did undergo; and
- *d*: the relative death rate (temporal death rate divided by temporal division rate);

then the plasmid is diluted/segregated at each division following the equation

$$F_{final}=F_{initial}\times (\frac{1+res}{2}{)}^{g}$$

So we can estimate

$$g=log_{2}(\frac{F_{final}}{F_{initial}})/log_{2}(\frac{1+res}{2})$$

Without any death, we would have

$$g_{no-death}=log_{2}(N_{final}/N_{initial})$$

The difference between the true number of generations *g* computed from plasmid frequency and this number of generations *g*_*no*−*death*_ computed based on the assumption that there is no death, allows us to estimate relative death rate as follows:

$$N_{final}=N_{initial}{\times 2}^{(1-d)*g}$$

This yields

$$d=1-\frac{log_{2}(N_{final}/N_{initial})}{g}$$

and thus

$$d=1-\frac{log_{2}(N_{final}/N_{initial})}{log_{2}(F_{final}/F_{initial})}\times log_{2}(\frac{1+res}{2})$$

The only remaining free parameter to estimate is *res*, which is estimated by performing growth kinetics without antibiotic treatment (in LB medium) and thus without (or with negligible) death. We then have

$$g=g_{no-death}$$

and thus

$$log_{2}(\frac{1+res}{2})=\frac{log_{2}(F_{final}/F_{initial})}{log_{2}(N_{final}/N_{initial})}$$

from which we can fit the value of the segregation parameter $log_{2}(\frac{1+res}{2})$ based on the values of *F* and *N* estimated by plating. Further experimental and mathematical details on the plasmid segregation system are given in S2–S5 Supporting Informations.

### Computing mutation rate taking death into account

Most modern measures of mutation rate rely on the same standard protocol, the fluctuation test [79], directly inspired by the Luria and Delbrück experiment [75]: several cultures are inoculated with a small population of nonmutant bacteria, are grown overnight and are then plated on selective media (to count the number of mutants in the final population) and on nonselective media (to count the total number of bacteria in the final population). The number of mutants *r* in the final population (or, rather, its distribution over several replicate populations) is used to estimate the number of mutational events *m* happening during growth. One should note that these two numbers are not equivalent, because one mutational event can lead to several mutants in the final population if it happens early during growth, making this part of the computation complicated for intuition, although good mathematical tools are available. The total number of bacteria *N* is assumed to be very close to the number of cell divisions (and thus the number of genome replications) because the initial number of bacteria is much smaller. Mutation rate can thus be estimated as *μ* = *m*/*N*.

The many existing software packages used to compute *m* from the observed distribution of *r* use an analytical expression of the probability-generating function (pgf) of the number of mutants in the final population [80]. The only free parameter is the number of mutational events (equivalent to the value of the mutation rate per division when scaled with population size). This parameter is estimated from plating data using the maximum likelihood principle. The most used implementation of this idea is FALCOR [39], available on a webpage: http://www.keshavsingh.org/protocols/FALCOR.html.

Other software packages implementing the same ideas have been developed more recently, including, for example, rSalvador [38] and bzRates [40], which also implement a few alternative assumptions such as fitness impact (cost or benefit) of the focal mutation or a more accurate correction for plating efficiency than the one suggested by FALCOR [81].

**Fig 10:**
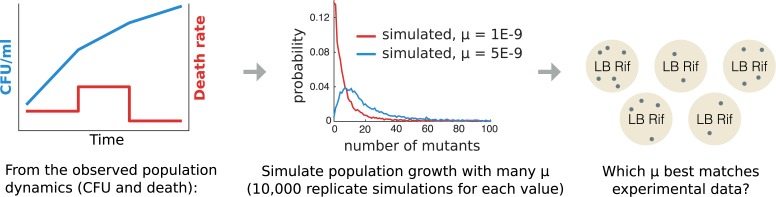
Using ATREYU to compute mutation rate from mutant counts and population dynamics. ATREYU takes as inputs the observed population dynamics (CFU counts at several time points and death rates between pairs of successive time points) and the count of mutants toward the phenotype of interest in several replicate cultures. Because it is based on simulation and does not require analytical expression of the mutant distribution, any nonstandard biological assumption (death, in our case) can easily be implemented. ATREYU, Approximate bayesian computing Tentative mutation Rate Estimator that You could Use; CFU, colony-forming unit.

However, to this day, no available software allows users to compute mutation rate when there is death. Some papers derived analytical expression of the pgf of the number of mutants in the final population in conditions in which there is death [82], but this has to our knowledge never been applied to real data nor implemented in a software package. In theory, such computations could easily be implemented in a tool similar to FALCOR (web server) or rSalvador (software package). However, the basic assumption of the derived formula is that death rate is constant. This assumption is the price to pay for an analytical expression for the pgf and is unfortunately not appropriate in our case, given the observed death kinetics (see Fig 3). On the other hand, given the computational power available today, we believe that analytical computations are not always necessary. In our case, while the measured population dynamics do not allow us to derive an analytical expression of the pgf, it is straightforward to simulate many times such population dynamics with an arbitrary mutation rate and to obtain an empirical distribution of the number of mutants. Running these simulations for any possible value of the mutation rate parameter then allows Bayesian inference: we look for the simulated mutation rate that gives the closest distribution to the one experimentally observed, as graphically represented in Fig 10. Such methods are classically referred to as Approximate Bayesian Computing. We implemented such simulations and inference in a Python software package, ATREYU, and use this software as the heart of our data analysis.

## Supporting information

S1 Supporting Information Detailed visualization of population dynamics for all biological replicates. Figure equivalent to Fig 3 of the main text, but with the behavior all biological replicates analyzed and represented independently.
(PDF)

S2 Supporting Information Measuring plasmid frequency: Mathematical assumptions and technical details. Experimental details on the use of the plasmid pAM34: dealing with plasmid copy number, appropriate quantity of IPTG, no satellite colonies observed.
(PDF)

S3 Supporting Information Testing the plasmid segregation approach to compute death rate. Estimating the accuracy of our estimation of death rate, using experiments in which death is mimicked with dilutions.
(PDF)

S4 Supporting Information Norfloxacin does not prevent plasmid replication in presence of the inducer. Norfloxacin is not likely to act directly on plasmid replication, because it does not cause plasmid segregation in presence of the inducer.
(PDF)

S5 Supporting Information Preparation of medium, strains, and reactants, and additional experimental details. Other experimental details: preparation of the strain, minimizing the variability between biological replicates by careful preparation and storage of reactants, behavior of the plasmid in a ΔlacY mutant and in presence of glucose.
(PDF)

## Abbreviations

ATREYU: Approximate bayesian computing Tentative mutation Rate Estimator that You could Use
CFSE: Carboxyfluorescein succinimidyl ester
CFU: colony-forming unit
FALCOR: Fluctuation AnaLysis CalculatOR
IPTG: Isopropyl β-D-1-thiogalactopyranoside
MIC: minimal inhibitory concentration
pgf: probability-generating function
