# Supplementary material for: Death and population dynamics affect mutation rate estimates and evolvability under stress in bacteria
Source: PLoS Biol. 2018 May 11;16(5):e2005056. doi: 10.1371/journal.pbio.2005056 (PMC5966242; doi:10.1371/journal.pbio.2005056)
Supplement: S1 Supporting Information — Figure equivalent to Fig 3 of the main text, but with the behavior all biological replicates analyzed and represented independently. (PDF) [file pbio.2005056.s001.pdf]

S1 Detailed visualisation of population dynamics for all biological replicates

In main text, figure 3 represents the average population dynamics obtained with several biological replicates. Here we represent independently the population dynamics obtained for each biological replicate, to convey the reader information about the variability inherent to this type of experiment.

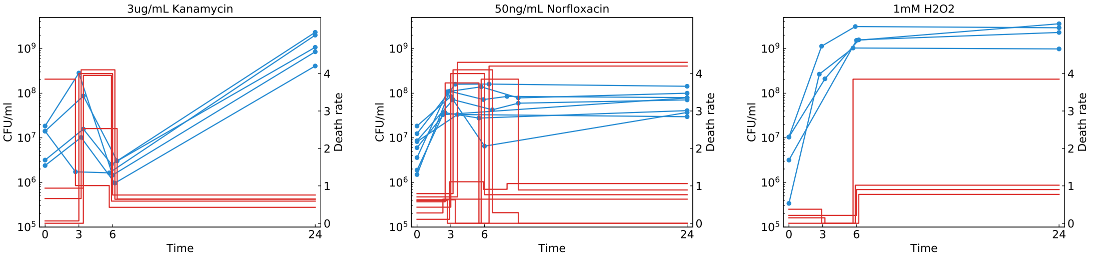

Growth and death dynamics of populations treated with sub-MIC antimicrobials. This figure is equivalent to figure 3 of the main text, but each line corresponds to a single biological replicate (comprising at least 6 replicate populations). Time points 3 and 6 hours were horizontally shifted by a small offset value to avoid overlapping lines. Death rates higher than 4 were set to 4, plus or minus a small offset value to avoid overlapping lines.
