# Supplementary material for: Death and population dynamics affect mutation rate estimates and evolvability under stress in bacteria
Source: PLoS Biol. 2018 May 11;16(5):e2005056. doi: 10.1371/journal.pbio.2005056 (PMC5966242; doi:10.1371/journal.pbio.2005056)
Supplement: S2 Supporting Information — Experimental details on the use of the plasmid pAM34: dealing with plasmid copy number, appropriate quantity of IPTG, no satellite colonies observed. (PDF) [file pbio.2005056.s002.pdf]

S2 Measuring plasmid frequency: mathematical assumptions and technical details

Our computations are based on plasmid frequency, which is obtained by dividing the number of bacteria bearing a copy of the plasmid (per unit of volume) by the total number of colony forming units (per unit of volume). In our segregation equations, we make the implicit assumption that bacteria that bear a copy of the plasmid only bear one. The copy number should be around 5-10 when the replication of the plasmid is fully induced, but our experiments have a pre-culture phase in which the plasmid starts segregating. Thus, when the treatment begins, the plasmid frequency in around 10%, and the fraction of bacteria carrying more than one copy should be negligible.

pAM34 bears a beta-lactamase, conferring resistance to ampicillin. Plasmid frequency is thus measured by plating on LB supplemented with 100ug/mL of ampicillin and 0.1mM of IPTG. The presence of IPTG is necessary for a bacteria bearing the plasmid to form a visible colony. We noticed that in solid medium, 1mM of IPTG is toxic and diminish the number of CFU (figure below). We thus use 0.1mM, which is a concentration that (1) is not toxic, and (2) is sufficient to allow all bacteria bearing the plasmid to form a visible colony, although the colonies grow slower than on LB due to imperfect plasmid replication.

Finally, we should note that the formation of satellite colonies is a phenomena frequently observed when plating on ampicillin to select beta-lactamase carrying bacteria. Satellite colonies are not resistant to ampicillin, but are sufficiently close to a resistant colony so that the beta-lactamase produced by this resistant colony deplete ampicillin from the growth medium. However in our setup, we do not observe any satellite colony. From 6 untreated cultures plated on LB ampicillin IPTG, we streaked 12 random colonies, 12 colonies very close to another colony, and 12 of the smallest colonies, on fresh agar plates of LB ampicillin IPTG, and confirmed that all these colonies where able to grow in isolation and thus genetically resistant to ampicillin.

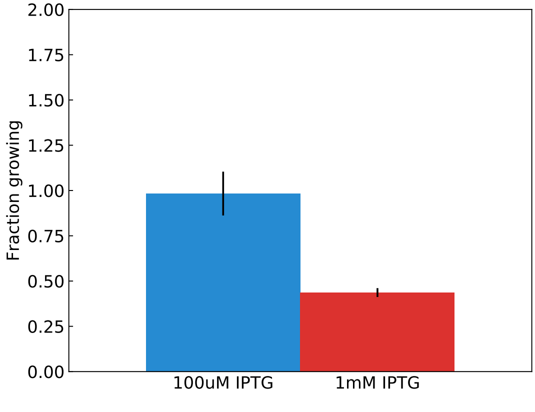

Toxicity of IPTG in solid medium at 1mM. A culture grown in LB supplemented with 1mM IPTG and 100ug/mL ampicillin (to ensure full maintenance of the plasmid) is diluted and plated on LB, LB + 1mM IPTG + 100ug/mL ampicillin, and LB + 0.1mM IPTG + 100ug/mL ampicillin. For both doses of IPTG in the solid medium, we plot the ratio of colony forming units in LB ampicillin IPTG and in LB. The ratio is much lower than 1 for 1mM IPTG, indicating toxicity, and close to 1 for 0.1 mM, indicating absence of toxicity.
