# Supplementary material for: Death and population dynamics affect mutation rate estimates and evolvability under stress in bacteria
Source: PLoS Biol. 2018 May 11;16(5):e2005056. doi: 10.1371/journal.pbio.2005056 (PMC5966242; doi:10.1371/journal.pbio.2005056)
Supplement: S3 Supporting Information — Estimating the accuracy of our estimation of death rate, using experiments in which death is mimicked with dilutions. (PDF) [file pbio.2005056.s003.pdf]

S3 Testing the plasmid segregation approach to compute death rate

We performed additional experiments in which 6 replicate populations are grown with the same protocol as for the rest of the article in untreated conditions (LB), but in which we dilute the culture after the initial time point to mimic the effect of death (a given fraction of of the population is effectively removed and replaced by fresh medium). Because we know the dilution, we know the equivalent death rate that our software should compute. We compare this expected death rate with the death rate measured from experimental data on the figure below.

Exp2 corresponds to a 100-fold dilution, and Exp3 to a 1000-fold dilution. Exp1 corresponds to a 10-fold dilution, but the same raw data are used in other computations to fit plasmid segregation parameters. The expected death rate equivalent to these dilutions is computed using the following formula (see main text for details).

$$d_{theoretical} = 1 - \frac{log_2(N_{final}/N_{initial})}{g}$$

The number of generations g is known from CFU counts and dilution factor:

$$g = log_2(N_{final}/N_{initial}) + log_2(dilution)$$

And thus we have:

$$d_{theoretical} = \frac{log_2(dilution)}{log_2(dilution) + log2(N_{final}/N_{initial})}$$

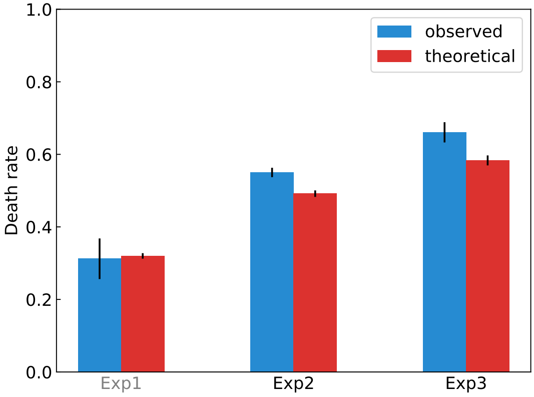

Diluting to mimic death. We compare the computed death rate with the expected one in experiments in which bacteria are diluted to mimic the effect of death.
