# Supplementary material for: Death and population dynamics affect mutation rate estimates and evolvability under stress in bacteria
Source: PLoS Biol. 2018 May 11;16(5):e2005056. doi: 10.1371/journal.pbio.2005056 (PMC5966242; doi:10.1371/journal.pbio.2005056)
Supplement: S4 Supporting Information — Norfloxacin is not likely to act directly on plasmid replication, because it does not cause plasmid segregation in presence of the inducer. (PDF) [file pbio.2005056.s004.pdf]

S4 Norfloxacin does not prevent plasmid replication in presence of the inducer

Some observations suggest that some quinolone antibiotics can cause plasmid segregation. We interpret this as an evidence that quinolone induced death in these experiments, however one may wonder whether their action on DNA topology may interfere with plasmid replication. We performed an experiment in which we treat plasmid-bearing bacteria with norfloxacin (at the same dose than in our other experiments), in presence of the inducer (1mM IPTG), and then plate on LB and on LB supplemented with 0.1mM IPTG to measure plasmid frequency. As shown on the figure below, plasmid frequency was maintained at 100%, indicating that norfloxacin does not prevent plasmid replication and that the segregation we observe in our experiments is not likely to be directly caused by norfloxacin via an action on plasmid DNA.

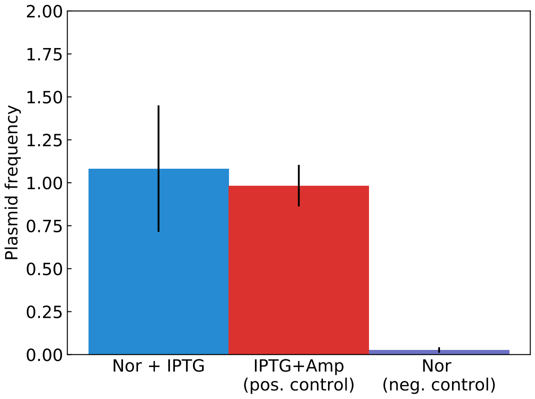

Norfloxacin does not cause plasmid segregation in presence of the inducer. We compare the fraction of cells retaining the plasmids after growth in 3 conditions: 50 ng/mL Norfloxacin + 1mM IPTG, 100ug/mL ampicillin + 1mM IPTG (positive control where the plasmid should be fully maintained), and Norfloxacin only (negative control, corresponds to the plasmid segregation we observe in absence of the inducer).
