# Supplementary material for: Death and population dynamics affect mutation rate estimates and evolvability under stress in bacteria
Source: PLoS Biol. 2018 May 11;16(5):e2005056. doi: 10.1371/journal.pbio.2005056 (PMC5966242; doi:10.1371/journal.pbio.2005056)
Supplement: S5 Supporting Information — Other experimental details: preparation of the strain, minimizing the variability between biological replicates by careful preparation and storage of reactants, behavior of the plasmid in a ΔlacY mutant and in presence of glucose. (PDF) [file pbio.2005056.s005.pdf]

S5 Preparation of medium, strains, and reactants, and additional experimental details

All reactants were prepared and used as detailed below. Aliquots containing the volume needed for a single biological replicate were prepared, so none of the reactants were frozen and unfrozen more than once. A fresh bottle of H_2O_2 was bought from Sigma for each biological replicate, stored at 4°C, and used within 7 days.

Reactant	Stock	Usage concentration
IPTG	1M in ddH2O, filtered, stored at -20°C	1mM (liquid), 0.1mM (agar)
Norfloxacin	10mg/mL in ddH2O, filtered, stored at -20°C	50ng/mL
Kanamycin	50mg/mL in ddH2O, filtered, stored at -20°C	3ug/mL
H_2O_2	1M in ddH2O, stored at 4°C	1mM
Ampicillin	100mg/mL in ddH2O, filtered, stored at -20°C	100ug/mL
Rifampicin	50mg/mL in DMSO, stored at -20°C	100ug/mL

LB growth medium was prepared following Miller recipe (10g/L Tryptone, 5g/M Yeast extract, 10g/L NaCl), in ddH2O. The medium was never stored more than a week. Agar plates supplemented with antibiotics were never conserved more than 2 days in the fridge (we noticed that on the time scale of a few weeks, IPTG degrades at 4°C, faster than ampicillin does).

The strain MG1655 pAM34 was prepared by electroporation of pAM34 (plasmid aliquot obtained by miniprep was a gift from W.-D. Hardt lab) in MG1655 (from the lab stock), followed by 1 hour of recovery in LB supplemented with 0.5mM IPTG, and plating on LB supplemented with 100ug/mL ampicillin and 0.1mM IPTG to select transformants. Hundreds to thousands transformants were routinely obtained from this method with no special difficulty, but particular attention should be given to let the cells recover in presence of IPTG but absence of ampicillin after electroporation, and to not use the glucose-containing SOC medium.

One of the initial difficulty we faced when working with this system was higher residual replication (in absence of the inducer) of the plasmid than expected. This residual replication is mathematically tractable, but probably increases the variability of our experiments. When supplementing the medium with 0.4%w/v glucose (to trigger catabolite repression of the plac promoter), we obtained lower values, similar to those reported by Gil and Bouché. We thus suspect that the LB medium used by Gil and Bouché contains glucose, which was common in 1991. In our case, the use of glucose is not desirable, because this would largely change the physiological state of the cells, their response to antibiotic stress, as well as their mutation rate and spectrum, preventing us from comparing our data with those obtained by other authors. We also tested the use of a ΔlacY deletion mutant, to prevent the import of traces of lactose that may be found in tryptone. However, the residual replication was not affected by this mutation, suggesting that lactose contamination is not a problem in our case. It should also be noted that lacY being a permease, its absence may affect membrane permeability and thus the response to antibiotic stress, which again is not desirable.
